# Rankl expression predicts poor prognosis in gastric cancer patients: results from a retrospective and single-center analysis

**DOI:** 10.1590/1414-431X20176265

**Published:** 2018-01-11

**Authors:** X. Zhang, Y. Song, N. Song, L. Zhang, Y. Wang, D. Li, Z. Wang, X. Qu, Y. Liu

**Affiliations:** 1 Department of Medical Oncology, The First Hospital of China Medical University, Shenyang, China; 2 Department of Surgical Oncology, The First Hospital of China Medical University, Shenyang, China; 3 Key Laboratory of Anticancer Drugs and Biotherapy of Liaoning Province, The First Hospital of China Medical University, Shenyang, China

**Keywords:** RANKL, RANK, Gastric cancer, IHC, overall survival

## Abstract

The receptor activator of nuclear factor κB ligand (RANKL)/RANK pathway plays an important role in the prognosis of several solid tumor types, but its role in gastric cancer prognosis has been poorly characterized. A total of 116 gastric cancer patients who underwent surgical resection were enrolled in this study. Expressions of RANKL and RANK in gastric cancer tissues were detected using immunohistochemical staining. Thirty-eight patients (33%) showed a high level of RANKL expression and 61 patients (53%) showed a high level of RANK expression. There was a positive correlation between expressions of RANKL and RANK (P=0.014, *r*=0.221). A high level of RANKL expression indicated shorter overall survival (OS) (P=0.008), and was associated with a higher pathological tumor/lymph node/metastasis (pTNM) stage (P=0.035), while no significant correlation was detected between RANK expression and clinicopathological parameters. RANKL also predicted poor prognosis in patients with high RANK expression (P=0.008) and Bormann's type III/IV (P=0.002). Furthermore, RANKL expression correlated with pTNM stage according to high RANK expression (P=0.009), while no significance was found in patients with low RANK expression (P=1.000). Together, our results revealed that high expression of RANKL could predict worse outcomes in gastric cancer especially combined with RANK detection, and thereby this pathway could be a useful prognostic indicator of gastric cancer.

## Introduction

Gastric cancer (GC) is the second most commonly diagnosed cancer and the second leading cause of cancer-related deaths in China (1), with the highest incidence rate in Eastern Asia. Even with radical surgery and chemotherapy, the prognosis of GC is still unsatisfactory (2,3). The failure of comprehensive therapies and poor prognosis in GC are due to the molecular complexity and heterogeneity of the disease (4,5). Diverse genetic factors play crucial roles in heterogeneity of GC (6,7). However, the genes that predict the progression of GC have not been systematically studied. Currently, serum biomarkers such as CEA, CA19-9 and CA72-4 lack sufficient sensitivity and specificity as prognostic predictors of GC (8,9). In addition to serum biomarkers, biomarkers from tumor specimens were also associated with prognosis, but there is still no specific prognostic biomarker for GC patients. Therefore, researchers are still investigating biomarkers that can monitor the progression of GC. This might allow a more appropriate patient stratification and provide guidance for new personalized treatments.

Receptor activator of nuclear factor κB ligand (RANKL), also known as TNFSF11, is a member of the tumor necrosis factor family of cytokines and is typically expressed on osteoblasts/bone stromal cells. RANKL and its receptor RANK, which is expressed on the surface of osteoclast precursors, are well known for their involvement in osteoclast survival, differentiation and activation (10,11). Expression of RANK was found in several solid cancer types such as breast, prostate, and hepatocellular carcinoma (12–14). Previous reports showed that the RANKL/RANK pathway was related to tumor progression and migration (especially bone metastasis) in breast cancer, prostate cancer, and lung cancer cells (12–15). Moreover, RANKL expression has been demonstrated in various cell types of normal tissues, including bone, brain, lymph nodes, mammary gland and thymic medulla (11,16–19). However, no study has examined the clinical significance of RANKL expression, RANK expression, and their prognostic value in GC.

In this study, we examined the expression of RANKL and RANK by immunohistochemistry (IHC) staining in tumor samples from 116 GC patients who underwent surgical treatment. The correlation between RANKL and RANK and their association with the clinicopathological characteristics and overall survival (OS) were also evaluated.

## Material and Methods

### Patients and tissue samples

Specimens of gastric adenocarcinoma tissue were collected from 116 patients who underwent D2 and R0 surgical resection at the First Affiliated Hospital of China Medical University between 2006 and 2011. Patients were retrospectively analyzed during the median follow-up of 34 (range 4-85) months from surgery. None of the patients received preoperative radiotherapy, chemotherapy or immunotherapy. Age, gender, pathological tumor/lymph node/metastasis (pTNM) stage, Lauren grade, Bormann's type and tumor location were assessed according to medical charts and pathology records. pTNM stage was evaluated following the 7th edition of American Joint Committee on Cancer Staging Manual. Lauren grade used was according to WHO classification. All the patients had been treated based on the latest guidelines; no patient received neo-adjuvant chemotherapy and radiotherapy. All research involving human participants was approved by the Ethics Committee of China Medical University. Written informed consents were obtained from all the participants in accordance with the Helsinki Declaration. Overall survival (OS) was defined as the time from the surgery until the time of death due to cancer or to last known follow-up.

### Immunohistochemistry analysis

Formalin-fixed, paraffin-embedded primary gastric carcinoma tissues were cut into 3-mm sections. The IHC method is discussed in our previous study (20). S-P immunohistochemical kit and 30-diamino-benzidine tetrahydrochloride (DAB) kit were obtained from Maixin Bio (Fuzhou Maixin Biological Technology Ltd., China). Immunohistochemical staining was performed using the following antibodies: anti-RANK antibody from RD Company and anti-RANKL antibody from Abcam (USA). Sections were observed through microscopy (×20 and ×40) by two independent pathologists. From each section, 5 visual fields were randomly selected and scoring was done according to the percentage of positive cells and the staining intensity. Positive cells of <10, 10-25, 26-50, 51-75, >76% were recorded as 0, 1, 2, 3, and 4, respectively. A score >2 was considered as high expression, 0-2 as low expression.

### Statistical analysis

The relationship between staining intensity and clinicopathological patterns was evaluated using Spearman rank correlation or χ^2^ test. The log-rank test and the Kaplan-Meier method were used for the patient survival analysis. P<0.05 was considered statistically significant. Statistical analysis was carried out using SPSS 21.0 software package (SPSS, Inc., USA).

## Results

### RANKL/RANK expression and clinicopathological parameters

**Figure 1.**
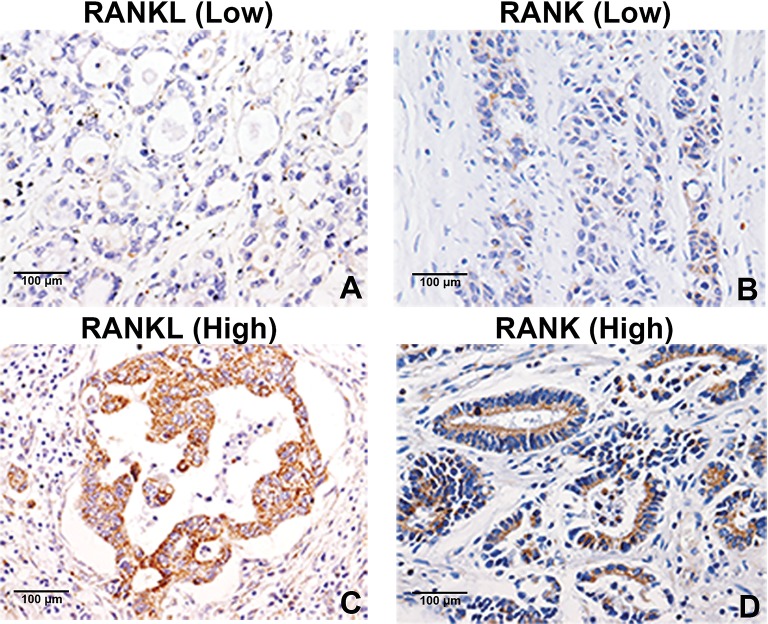
Expression of RANKL and RANK in gastric cancer tissues. RANKL (*A*) and RANK (*B*) low staining levels; RANKL (*C*) and RANK (*D*) high staining levels (in brown). Magnification ×400.

High RANKL expression was detected in 38 (33%) and 61 patients (53%). Figure 1 shows representative images of low and high RANKL/RANK expression. Correlations between expression of RANKL, RANK and clinical characteristics of primary GC patients are given in Table 1. We found that more patients with high RANKL expression were at late pTNM stage (P=0.035). However, no association was found between RANKL expression and age, gender, T stage, N stage, Lauren grade, Bormann's type or tumor location (all P>0.05). Additionally, no association was found between RANK expression and characteristics of primary GC patients (all P>0.05). The relationship between expression of RANKL and RANK in GC patients was further investigated. We found a weak positive correlation between the expression of RANKL and RANK (P=0.014, *r*=0.221; Table 2).

**Table 1.** Correlation between the expression of RANKL/RANK and the clinicopathological factors in primary gastric cancer patients.

Characteristics	RANKL			RANK		
	Low (%)	High (%)	P value	Low (%)	High (%)	P value
Gender			0.363			0.280
Male	57 (65)	31 (35)		39 (44)	49 (56)	
Female	21 (75)	7 (25)		16 (57)	12 (43)	
Age (years)			0.074			0.575
<60	39 (60)	26 (40)		29 (45)	36 (55)	
≥60	39 (76)	12 (24)		26 (51)	25 (49)	
pTNM stage			0.035*			0.508
I+II	22 (85)	4 (15)		14 (54)	12 (46)	
III	56 (62)	34 (38)		41 (46)	49 (49)	
T stage			0.541			0.140
T1-2	10 (77)	3 (23)		9 (69)	4 (31)	
T3-4	68 (66)	35 (34)		46 (45)	57 (55)	
N stage			0.321			0.816
N0	18 (78)	5 (22)		10 (44)	13 (56)	
N1-3	60 (64)	33 (36)		45 (48)	48 (52)	
Lauren grade			0.429			0.215
Intestinal	33 (70)	14 (30)		27 (57)	20 (43)	
Diffuse	30 (68)	14 (32)		16 (36)	28 (64)	
Mixed	15 (60)	10 (40)		12 (48)	13 (52)	
Bormann's type			0.541			0.565
I+II	10 (77)	3 (23)		5 (39)	8 (61)	
III+IV	68 (66)	35 (34)		50 (49)	53 (51)	
Location			0.673			0.173
Upper one-third	8 (67)	4 (33)		5 (42)	7 (58)	
Middle one-third	7 (64)	4 (36)		7 (64)	4 (36)	
Lower one-third	54 (70)	23 (30)		39 (51)	38 (49)	
≥2 areas	9 (56)	7 (44)		4 (25)	12 (75)	

Data are reported as numbers and percentages. pTNM: pathological tumor/lymph node/metastasis.

* P<0.05 (Spearman rank correlation or χ^2^ test).

**Table 2.** Spearman's correlation between RANKL and RANK expression in primary gastric cancer patients.

RANKL	Cases (%)	RANK		Spearman's r	P value
		Low (%)	High (%)		
Low (%)	78 (67)	43 (55)	35 (45)	0.221	0.014*
High (%)	38 (33)	12 (32)	26 (68)		
Total (%)	116 (100)	55 (47)	61 (53)		

Data are reported as number and percentage.

* P<0.05 (χ^2^ test).

### RANKL/RANK expression and OS

**Figure 2.**
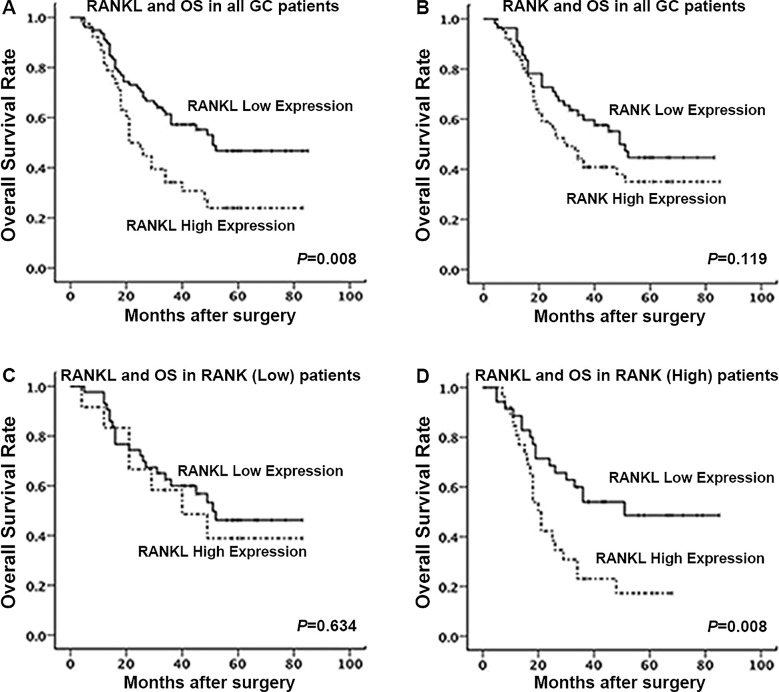
Prognostic significance of RANKL and RANK expression in gastric cancer (GC) patients. Kaplan-Meier analysis of overall survival (OS) for RANKL (*A*) and RANK (*B*) expression in all gastric cancer patients. Stratification analysis for the RANKL expression in low (*C*) and high (*D*) RANK expression patients.

To determine the prognostic value of RANKL/RANK expression, survival analysis was performed. All patients were followed up until July 2014. Among the total 116 patients, 66 patients (57%) died during follow-up. Kaplan-Meier analysis revealed that the OS was longer for patients with low RANKL expression than those with high RANKL expression (P=0.008, Figure 2A). No significant association was found between RANK expression and OS (P=0.119, Figure 2B). We next divided patients into two groups according to the expression level of RANK and found that RANKL expression significantly correlated with OS in the high RANK group (P=0.008, Figure 2C), while no significant association was observed in the low RANK group (P=0.634, Figure 2D). In univariate analysis using COX proportional-hazard models, male, advanced pTNM stage, advanced T stage, presence of lymph node metastasis, tumor in more than one-third of gastric area, and enhanced RANKL expression were associated with reduced OS (Table 3). The further multivariate COX analysis identified RANKL to be an independent predictor of poorer OS (HR=1.687, P=0.045; Table 3).

**Table 3.** Univariate and multivariate analyses of overall survival according to clinicopathological factors and RANKL expression.

Characteristics	Cases		Univariant analysis		Multivariant analysis	
	Patients	Deaths	P value	HR	P value	HR
Gender			0.090	0.571	0.345	0.725
Male	88	55				
Female	28	11				
Age (year)			0.184	1.387		
<60	65	33				
≥60	51	33				
pTNM stage			0.002	3.450	0.844	1.162
I+II	26	7				
III+IV	90	59				
T stage			0.010	6.370	0.105	4.349
1-2	13	2				
3-4	103	64				
N stage			0.022	2.379	0.471	1.577
0	23	8				
1-3	93	58				
Lauren grade			0.863	1.028		
Intestinal	47	26				
Diffuse	44	24				
Mixed	25	16				
Bormann's type			0.117	1.962		
I+II	13	6				
III+IV	103	60				
Location			0.040	1.464	0.074	1.387
Upper one-third	12	7				
Middle one-third	11	4				
lower one-third	77	42				
≥2 areas	16	13				
RANKL			0.009	1.916	0.045	1.687
Low	78	38				
High	38	28				

Data are reported as numbers. HR: hazard risk; pTNM: pathological tumor/lymph node/metastasis.

### Clinicopathological parameters in patients with different RANK expression

Among patients with high RANK expression, those with high RANKL expression were more likely to have higher pTNM stage (P=0.009), while no significance was found with RANKL expression and pTNM stage in patients with low RANK expression (P=1.000). RANKL expression was associated with Lauren grade in patients with low RANK expression (P=0.038), but not in patients with high RANK expression (P=0.091) (Table 4).

**Table 4.** Correlation between the expression of RANKL and the clinicopathological factors in patients with different RANK expression.

Characteristics	RANK (low, %)			RANK (low, %)		
	RANKL low	RANKL high	P value	RANKL low	RANKL high	P value
Gender			1.000			0.532
Male	30 (77)	9 (23)		27 (55)	22 (45)	
Female	13 (81)	3 (19)		8 (67)	4 (33)	
Age (years)			0.108			0.438
<60	20 (69)	9 (31)		19 (53)	17 (47)	
≥60	23 (89)	3 (11)		16 (64)	9 (36)	
pTNM stage			1.000			0.009*
I+II	11 (79)	3 (21)		4 (100)	0 (0)	
III	32 (78)	9 (22)		31 (54)	26 (46)	
T stage			0.392			0.129
T1-2	6 (67)	3 (33)		9 (69)	4 (31)	
T3-4	37 (80)	9 (20)		46 (45)	57 (55)	
N stage			1.000			0.128
N0	8 (80)	2 (20)		10 (77)	3 (23)	
N1-3	35 (78)	10 (22)		25 (52)	23 (48)	
Lauren grade			0.038*			0.091
Intestinal	25 (93)	2 (7)		8 (40)	12 (60)	
Diffuse	10 (63)	6 (37)		20 (71)	8 (29)	
Mixed	8 (67)	4 (33)		7 (54)	6 (46)	
Bormann's type			1.000			0.448
I+II	4 (80)	1 (20)		6 (75)	2 (25)	
III+IV	39 (78)	11 (22)		29 (55)	24 (45)	
Location			0.967			0.917
Upper one-third	4 (80)	1 (20)		4 (57)	3 (43)	
Middle one-third	5 (71)	2 (29)		2 (50)	2 (50)	
Lower one-third	31 (80)	8 (20)		23 (61)	15 (39)	
≥2 areas	3 (75)	1 (25)		6 (50)	6 (50)	

Data are reported as numbers and percentages. pTNM: pathological tumor/lymph node/metastasis.

* P<0.05 (Spearman rank correlation or χ^2^ test).

**Figure 3.**
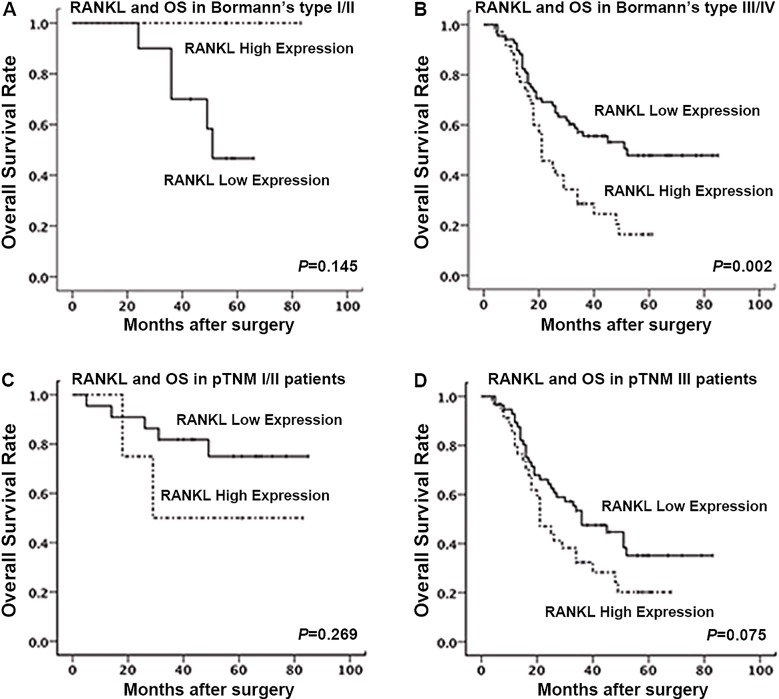
Prognostic significance of RANKL expression according to different clinicopathological factors in gastric cancer patients. Kaplan-Meier analysis of overall survival for the RANKL expression in Bormann's type I/II (*A*) or III/IV (*B*) patients, and for pTNM stage I/II (*C*) or III (*D*) patients. pTNM: pathological tumor/lymph node/metastasis.

Next, we explored the prognostic significance of RANKL expression in GC patients with Bormann's type and pTNM stage. While no significant correlation was found in Bormann's type I/II (P=0.145; Figure 3A), we found that RANKL was significantly correlated with OS in Bormann's type III/IV (P=0.002; Figure 3B). Since the expression of RANKL in GC patients was associated with pTNM stage, we further performed prognostic analysis between RANKL expression and OS in patients with pTNM stage I/II and III using Kaplan-Meier analysis. The result showed that RANKL expression was not correlated with OS in either pTNM stage I/II (P=0.269; Figure 3C) or III (P=0.075; Figure 3D) groups.

## Discussion

Previous studies showed that the RANKL/RANK pathway is associated with bone metastasis. Increasing number of studies have shown a positive relationship between this pathway and tumorigenesis (20,21) and visceral metastasis (22–24) in several malignant tumors, and analyses of RANKL expression in various tumors have proven that RANKL plays an important role in cancer progression and metastasis (25,26). A study by Hofman et al. (27) revealed that RANKL mRNA levels were abundantly expressed in tissues of 69 GC patients. The expression of RANK, the only receptor of RANKL identified thus far (28,29), was also detected in various solid tumors (15,30–32). However, the prognostic value of RANKL/RANK expression in GC had not been studied. Our study showed a negative relationship between RANKL and OS in GC patients, while no correlation between RANK and OS was found.

In the present study, high RANKL expression was observed in 38 of 116 patients (33%) with poorer prognosis. Moreover, RANKL expression significantly correlated with pTNM stage in GC patients. We identified high RANK expression in 61 patients (53%). RANK has been considered a predictive factor for prognosis and metastasis in several malignancies. However, our findings revealed no association between RANK expression and either OS or clinical characteristics of GC patients. The patients in our study were mostly with advanced stage cancer; hence, larger cohorts of patients are required to confirm our observations. We also found a positive correlation between RANKL expression and RANK expression.

Stratification analysis showed that the prognostic significance of high RANKL expression was duplicated in patients with high RANK expression, whereas in the patients with low RANK expression, RANKL expression had no impact on survival. This showed that higher RANKL and RANK expressions might predict the worst survival. The stratification analysis suggested that RANKL expression was more relevant to pTNM stage in patients with high RANK expression than in patients with low RANK expression. RANKL expression was closer to Lauren grade in patients with low RANK expression than those with high RANK expression. Moreover, we found a poorer survival with high RANKL expression in Bormann's III/IV patients than in Bormann's I/II patients, proving the predictive value of RANKL in Bormann's III/IV GC patients. Moreover, there was no significant correlation between RANKL expression and OS in either pTNM I/II or III patients.

To our knowledge, the present study is the first to assess the expression of RANKL and RANK in GC patients and to determine their prognostic significance. High RANKL expression, especially combined with high RANK expression, may be a negative prognostic biomarker in GC patients. Therefore, the RANKL/RANK pathway may be a potential target for developing novel anti-cancer therapies. It may be possible that the RANKL/RANK pathway activates NF-κB signaling to promote GC progression, as shown in previous studies in osteoclast survival (33). Additional studies are needed to further evaluate the specific mechanisms involved in this relationship, and larger multi-institutional prospective studies are necessary to support our results.
